# Orchestration of Angiogenesis by Immune Cells

**DOI:** 10.3389/fonc.2014.00131

**Published:** 2014-07-02

**Authors:** Antonino Bruno, Arianna Pagani, Laura Pulze, Adriana Albini, Katiuscia Dallaglio, Douglas M. Noonan, Lorenzo Mortara

**Affiliations:** ^1^Scientific and Technology Pole, IRCCS MultiMedica, Milan, Italy; ^2^Department of Biotechnology and Life Sciences, University of Insubria, Varese, Italy; ^3^Department of Research and Statistics, IRCCS Arcispedale Santa Maria Nuova, Reggio Emilia, Italy

**Keywords:** inflammation, angiogenesis, angiogenic switch, immune cells

## Abstract

It is widely accepted that the tumor microenvironment (TUMIC) plays a major role in cancer and is indispensable for tumor progression. The TUMIC involves many “players” going well beyond the malignant-transformed cells, including stromal, immune, and endothelial cells (ECs). The non-malignant cells can acquire tumor-promoting functions during carcinogenesis. In particular, these cells can “orchestrate” the “symphony” of the angiogenic switch, permitting the creation of new blood vessels that allows rapid expansion and progression toward malignancy. Considerable attention within the context of tumor angiogenesis should focus not only on the ECs, representing a fundamental unit, but also on immune cells and on the inflammatory tumor infiltrate. Immune cells infiltrating tumors typically show a tumor-induced polarization associated with attenuation of anti-tumor functions and generation of pro-tumor activities, among these angiogenesis. Here, we propose a scenario suggesting that the angiogenic switch is an immune switch arising from the pro-angiogenic polarization of immune cells. This view links immunity, inflammation, and angiogenesis to tumor progression. Here, we review the data in the literature and seek to identify the “conductors” of this “orchestra.” We also suggest that interrupting the immune → inflammation → angiogenesis → tumor progression process can delay or prevent tumor insurgence and malignant disease.

## Introduction

Tumors are tissues: the mass of most solid tumors contains a significant portion of untransformed host cells and matrix components in addition to transformed tumor cells. Within the “concert hall” of a tumor there is an extremely heterogenic “orchestra” where numerous factors interplay with each other at the cellular and molecular levels, to create a sort of symphony known as the tumor microenvironment (TUMIC). The TUMIC generally includes a broad array of immune and inflammatory cells as well as stromal and endothelial cells (ECs). These cell types are able to develop a dynamic, often tumor-promoting function at all stages of carcinogenesis (1).

The links between cancer and angiogenesis as well as between cancer and inflammation have been extensively documented. Angiogenesis is a crucial event for cancer survival and progression, since the vascular system delivers nutrients and oxygen to cancer cells, as well as furnishing the “roadways” through which transformed cells can invade distant organs and tissues.

In the 1800s, the observation that most tumors contain numerous inflammatory leukocytes led the pathologist Rudolph Virchow to suggest a functional relationship between chronic inflammation and cancer. However in the nineteenth century (2), tumor infiltrating immune cells were considered an attempt of the immune system to reject the tumor. Only in the last three decades the role of immune cells in promoting tumor progression has come back into light (2–6). Immune cells can act against tumors through direct and indirect mechanisms, potentially leading to tumor eradication, or resulting in immuno-editing of tumors (7). Both innate and adaptive immune cells can show strong anti-tumor activities. When altered, this functional relationship plays a crucial role in inducing and shaping tumor angiogenesis, inhibiting anti-tumor immune responses, and promoting a favorable microenvironment in which tumor cells can survive and replicate. Within the TUMIC immune cells can be considered an orchestra conductor of a major symphony: on one hand they can directly or indirectly destroy cancer cells, on the other hand they may promote tumor growth and dissemination (4, 5). This “immunologic switch” within the TUMIC that can promote or inhibit tumor formation and progression to malignancy is largely due to different polarization states of the immune cells. A classic example of polarization is that of T cells and macrophages. T helper 1 (Th1) cells are programed to exert cellular cytotoxicity; by analogy, M1 macrophages are classically activated cells producing Th1 cytokines involved in acute inflammatory responses and potential cytotoxicity. While Th2 cells are skewed toward humoral immunity, M2 polarized macrophages show a phenotype associated with Th2 cytokines, tissue reconstruction, growth promotion, and angiogenesis (4, 5, 8). Recent evidence suggests that many immune cell subsets show diverse polarization states, particularly within the TUMIC (4, 5, 8, 9). Both local and systemic immune polarization, at least in part, explains the difficulty in translating promising immunotherapy approaches into the clinic, in spite of intensive efforts over numerous years. While the anti-tumor potential of immune cells has been extensively reviewed and will be discussed elsewhere in this issue, here we focus on the pro-tumor activities of immune cells, in particular on angiogenesis and selected mechanisms associated with pro-tumor polarization.

This “orchestration” of tumor angiogenesis, driven by immune cells, can be considered a common feature both for solid and hematologic malignancies. It therefore represents a valid target for anti-tumor therapies and cancer preventive strategies.

## Macrophages

Macrophages are immune cells recruited in response to tissue damage and inflammation, acting as “professional” phagocytic cells specialized in the clearance of pathogens and antigen presentation to the adaptive immune system. Macrophages undergo activation to various polarization states on the basis of the signals coming from the surrounding microenvironment. M1 macrophages produce significant quantities of pro-inflammatory cytokines, mediate resistance against pathogens, and can kill tumor cells. M1 macrophages are generally characterized by an interleukin (IL)-12^high^, IL-23^high^, IL-10^low^ phenotype, driving Th1 response; they can also produce reactive oxygen and nitrogen species (ROS and NOS) (8, 10–13).

**Figure 1:**
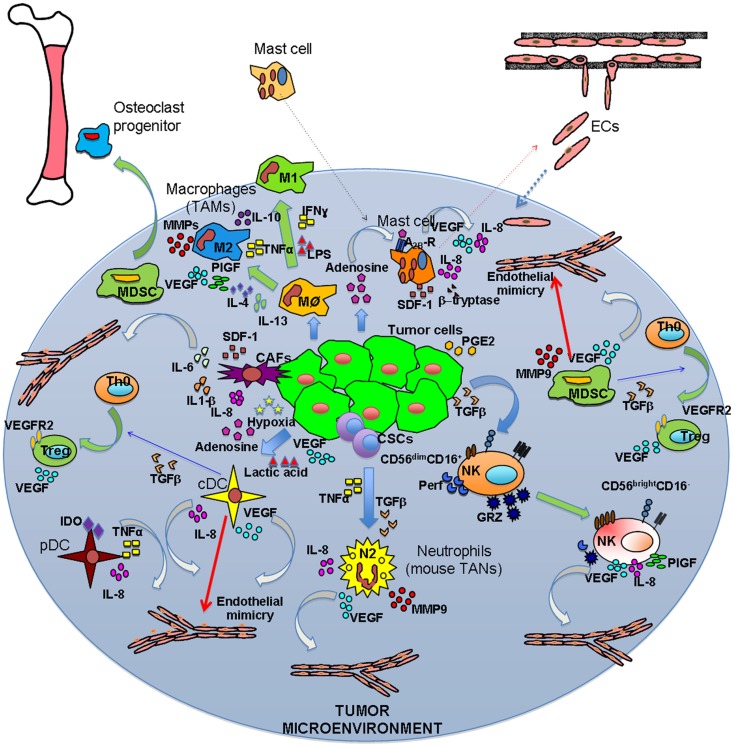
**Inflammatory orchestration of tumor angiogenesis**. Both solid and hematological malignancies are associated with an inflammatory state characterized by different innate and adaptive immune cells. These cells are able to play two different symphonies at the same: on one hand they can contribute to tumor suppression and eradication, on the other they play a key role in tumor insurgence and progression. The TUMIC produces several factors, including TGFβ, PGE2, VEGF, lactic acid, and adenosine, which contribute to polarization of immune cells toward a pro-tumor/pro-angiogenic phenotype. Polarization is not only mediated by tumor cell products, but also involves crosstalk between immune cells. Ag–Ab, antigen–antibody complexes; CSCs, cancer stem cells.

M2 activation is closely related to the tumor-associated macrophage (TAM) profile (8). *In vitro*, M2 polarization can be obtained by treating cells with specific cytokines/immune stimulants resulting in the generation of different cellular subsets. For example, the anti-inflammatory M2a phenotype is produced by IL-4 and IL-13, the M2b phenotype is generated in response to immune complexes and toll-like receptor (TLR)/IL-1 receptor ligands; finally, the M2c phenotype is induced in the presence of IL-10. M2a polarized macrophages produce Th2 cytokines with an IL-12^low^, IL-23^low^, IL-10^high^ phenotype. Overall, M2 macrophages promote tissue remodeling and angiogenesis (8, 12, 13). TAMs show a similar molecular profile (Figure 1), influencing angiogenesis, invasion, and metastasis (8), as well as subversion of adaptive immunity (13). Both M1 and M2 macrophages are recruited into tumors from circulating blood monocytes by chemokines, but they can also migrate from adjacent tissues (8).

## Tumor-Associated Macrophages

Tumor-associated macrophages are largely derived from peripheral blood monocytes recruited into the tumor mass by growth factors and chemokines. Among these are CCL2, vascular endothelial growth factor (VEGF), and the molecules involved in the CXCL12/CXCR4 signaling axis (8, 14, 15). TAMs are recruited into and tend to accumulate in necrotic areas where they remove the tissue debris and stimulate repair processes (16–19). In keeping with this, higher numbers of TAMs are found in tumors with extensive necrosis as compared to those with limited necrotic areas (20). In addition, hypoxia stimulates production of VEGF and CXCL12 by both tumor and normal cells, these factors are associated with M2 polarization (8, 14, 15). Hypoxia stimulates TAMs to co-operate with tumor cells in promoting revascularization (21, 22). Key players in response to hypoxia are the hypoxia-inducible factor (HIF)-1α and HIF-2α, the latter of which is expressed in a more tissue-restricted manner. Although they have extensive sequence and functional similarity, these two molecules show several differences and even opposing activities in some cases. Macrophages cultured under hypoxic conditions express HIF-1α and HIF-2α both *in vitro* and *in vivo* (23, 24). However, under these conditions, they express higher levels of HIF-1α as compared to HIF-2α, and consistent with these findings, the levels of HIF-1α are higher in TAMs infiltrating breast and ovarian carcinomas (23). HIF-2α is expressed in human cancers and correlates with poor prognosis (25–27). Murine myeloid specific knockouts of both HIF-1α and HIF-2α show distinct activities in regulating the immune response. Mice lacking myeloid HIF-1α show reduced migration and invasion of macrophages, limited acute inflammation, and inhibition of bactericidal activity (28–30). Mice lacking myeloid HIF-2α are resistant to endotoxemia and inflammatory lesions (31). Further, they showed resistance in a colitis associated colorectal cancer model and fewer macrophages infiltrating the tumors (31).

Tumor-associated macrophages can promote angiogenesis through numerous mechanisms (Figure 1), in particular by producing pro-angiogenic factors and inducing degradation of the extracellular matrix (ECM). Among the pro-angiogenic factors produced by TAMs are VEGF, EGF, members of the FGF family which are able to stimulate the recruitment and migration of ECs, PDGF-B, also implicated in pericyte recruitment, angiogenic CXC chemokines (CXCL8/IL-8 and CXCL12, also known as stromal derived factor-1, SDF-1), and angiogenesis-associated factors such as transforming growth factor beta (TGFβ), tumor necrosis factor alpha (TNFα), and thymidine phosphorylase (8, 15). TAM-derived cytokines can also act on angiogenesis in an indirect manner by autocrine stimulation of TAM activity.

Moreover, TAMs release different proteases, including matrix metalloproteinases (MMPs 1, 2, 3, 9, and 12), as well as plasmin and urokinase plasminogen activator, whose combined action induces degradation of the basement membrane and ECM components, destabilization of the vasculature as well as migration and proliferation of ECs (8, 15, 21). This co-operation facilitates the migration and extravasation of tumor cells during the metastatic process (32).

Tie2-expressing macrophages (TEMs) represent a TAM subset closely associated with the vasculature (33, 34). These cells appear to have a distinct gene signature (35) in spite of substantial overlaps between TAMs, TEMs, myeloid-derived suppressor cells (MDSCs), monocytes, and embryonic/fetal macrophages (35, 36). TEMs are also recruited at the tumor site after treatment with vascular disrupting agents, interfering with and antagonizing their action (37). This suggests that TEMs could be key targets for anti-angiogenic therapy; deletion of TEMs inhibits angiogenesis and tumor growth (33, 34, 38). TEMs are likely to be among the myeloid cells associated with generation of the pre-metastatic niche. The pre-metastatic niche consists in the preparation of a hospitable local microenvironment that can be easily seeded by circulating tumor cells. Diverse myeloid cells are clearly involved in generation of the pre-metastatic niche (8, 39–42), which appears to be a key factor in metastatic dissemination. Targeting the angiopoietin (Ang)2/Tie2 axis by blocking Ang2 resulted in inhibition of Tie2 up-regulation in TAMs (43), and inhibits vessel destabilization (44), thus influencing the pre-metastatic niche and inhibiting metastatic dissemination (45).

## Neutrophils

Neutrophils are the most abundant human leukocytes and play a key role in innate immunity, representing the first immune cell recruited into sites of infection. In response to several stimuli, they are quickly recruited into areas producing “danger signals,” where they employ strategies, based mainly on pattern recognition mechanisms, to contain and clear infection. Among the response mechanisms, a key player is neutrophil degranulation, leading to the release of lytic enzymes, as well as respiratory burst production of ROS (${\text{O}}_{2}^{-}$, H_2_O_2_, HOCl) with antimicrobial potential (46). Further, neutrophils are also the source of several cytokines, including TNFα, IL-1β, IL-1Rα, IL-12, and VEGF and chemokines such as CXCL1, CXCL8, CXCL9, CXCL10, CCL3, and CCL4 (47) directly involved in tissue reconstruction and angiogenesis. Neutrophils have been shown to be required for vascularization of the endometrium (48, 49).

Neutrophils can infiltrate tumor tissues (Figure 1), having been observed in colon adenocarcinoma, myxofibrosarcoma, gastric carcinoma, and melanoma, suggesting a potential role in tumor progression and angiogenesis (50). In patients with myxofibrosarcoma, enhanced neutrophil number correlates with increased intra-tumor microvessel density (51). Interestingly, CXCL8, which is abundantly produced by tumor cells, is released in the surrounding environment, representing a potent chemoattractant for neutrophils within the tumor mass. CXCL8 and other “ELR” CXC chemokines have been associated with angiogenesis by direct activation of CXCR2 on ECs (52). However, only a subset of ECs expresses CXCR2 (53, 54). *In vivo* neutrophils are required for angiogenesis induced by CXCR2 ligands in the matrigel sponge model (55), yet are not necessary in the corneal pocket assay (52), suggesting that endothelial subtypes may be variably responsive to CXCR2 ligands, while neutrophils are uniformly responsive to these molecules. In a ras oncogene driven tumor progression model, tumor-associated neutrophils (TANs) mediate IL-8-induced angiogenesis (56, 57). The fact that angiostatin, an angiogenesis inhibitor identified *in vivo* (58), effectively targets monocytes, macrophages, and neutrophils (55, 59–64) clearly suggests that these cells play a key role in this process.

Activated neutrophils can release a variety of proteases that can degrade and remodel the ECM (Figure 1), in particular MMP9. Neutrophil-derived MMP9 has been found to be important in models of skin and rip-Tag pancreatic cancers (1) where they sustain tumor angiogenesis. TNFα, a cytokine released into the TUMIC and linked to tumor progression (65), induces neutrophil degranulation and VEGF release (66) and CXCL8, CXCL1 production (67), thus favoring angiogenesis.

Neutrophils have also been reported to produce angiostatin itself (68) and are associated with anti-angiogenic tumor repression in peroxisome proliferator-activated receptor alpha (PPARα) deficient mice (69). The pro- and anti-angiogenic activities of neutrophils, and their role in tissue destruction or reconstruction, suggest that subsets of neutrophils characterized by different activities may exist (5, 70). Experimental evidence in murine models supports this hypothesis (71–73). When TGFβ activity was blocked, anti-tumor “N1” neutrophils were found to be associated with direct tumor cell killing as well as activation of CD8^+^ T cells. In control animals, pro-tumor “N2” neutrophils were instead observed (70), indicating a role for TGFβ. Depletion of neutrophils under the TGFβ blockade impaired CD8^+^ T cell activation and enhanced tumor growth, while in control animals TANs depletion resulted in slower growth and increased CD8^+^ activation (Figure 1). However, to date little is known concerning the existence and eventual role of neutrophil subsets in humans.

## Dendritic Cells

Dendritic cells (DCs) are fundamental innate immune cells with a key role in priming, orientation, and regulation of adaptive immune responses (74). DCs represent a heterogeneous population, including two major cell types: conventional (or myeloid) DCs (cDCs) and plasmacytoid DCs (pDCs) (75). They act as sentinels in the periphery, and after recognizing and capturing microbial antigens, they migrate in the secondary lymphoid organs, process foreign antigens and present peptide epitopes to naïve T lymphocytes, acting as potent antigen-presenting cells (APCs) (76). Both DCs subsets become fully mature following stimulation, typically in response to invading microbial pathogens, to become APCs. The cDCs mainly secrete IL-12, while pDCs release interferon (IFN)α (77, 78). However, in an immature state, they function as tolerance-inducing cells, impeding and regulating the activation of pool of latent and auto-reactive T cells and autoimmunity. Since DCs play a key role in T cell responses to antigens, several preclinical and clinical studies have been addressed to reinforce their APC function, in order to enhance anti-tumor T immune responses (79–84). DCs represent another immune cell type that could be altered in its “conventional” function by tumor cells and the TUMIC (75, 85–89), thus contributing to the inflammatory “orchestration” of tumor angiogenesis.

Clinical studies have shown that in diverse tumor types, DCs display specific alterations in their stimulatory capacity, and the host can develop anomalous myeloid cell differentiation (90–96). One of the mechanisms driving this abnormal myeloid cell differentiation is the constitutive activation of signal transducers and activator of transcription-3 (STAT3) that promotes the continuous proliferation and accumulation of immature myeloid cells, including DCs, thus contributing to the suppression of tumor-specific immune responses (97). STAT3 signaling in myeloid cells has been associated with angiogenesis (98). Potential therapeutic strategies might include inhibition of STAT3 signaling (97). Soluble factors, such as VEGF, IL-6, and TGFβ, can contribute to reduction of mature DC numbers, expansion and accumulation of immature tolerant DCs, and eventual polarization of DCs toward Th2 or T regulatory (Treg) induction, all features that contribute to tumor evasion from immune response. These features, described in both cancer patients and in tumor-bearing animals, have lead to the definition of a new DC cell subtype, termed regulatory DCs (regDCs) (99, 100). These tumor-associated DCs are potent immune-suppressive cells with different phenotypes and functions, including pDCs, cDCs, and also MDSCs, as it has very recently been described in a Lewis Lung (LL3) mouse model (101).

In human ovarian carcinomas, CXCL12-recruited pDCs have been shown to produce TNFα and IL-8 (Figure 1), favoring tumor angiogenesis (102). Tumor-conditioned pDCs can also act as potent immuno-suppressive cells, exerting a strong reduction of an efficient immune response (103–105). Human ovarian cancer-derived DCs co-express EC and DC markers and can therefore also significantly influence tumor angiogenesis by trans-differentiating into endothelial-like cells (Figure 1), thus promoting the formation of fully functional blood vessels (106).

In their immature state, DCs acquire the ability to trigger and guide development of Treg cells (Tregs) through TGFβ production (Figure 1), inducing a tolerogenic TUMIC (107–110). Hypoxia, which represents a key feature in the TUMIC, together with adenosine release severely inhibits DC migratory capacity (111, 112). In hypoxia, DCs are polarized to a Th2-stimulating phenotype (113). DCs differentiated in the presence of adenosine express higher levels of VEGF, IL-6, IL-8, IL-10, COX2, TGFβ, and indoleamine 2,3-dioxygenase (IDO) (114), thus sustaining tumor angiogenesis. DCs alternatively activated with IL-10, calcitriol, and prostaglandin (PG) E2, acquire pro-angiogenic activities (115). Interestingly, many tumor-derived soluble factors, like VEGF (116), adenosine (117), PGE2 (118), and TGFβ (119), which are crucial for EC activation, migration, and functionality, are also able to mediate inhibitory effects on DC activation, resulting in T cell suppression and induction of Tregs. The strong alteration and inhibition in antigen presentation and DC maturation observed in cancer patients is thought to be mainly due to VEGF (120–122). Moreover, tumor-associated cDCs express high levels of the programed cell death ligand 1 (PDL1), an important negative-regulatory ligand that suppresses T cell activation, in response to tumor-derived VEGF (102).

Two pro-inflammatory molecules released by DCs, TNFα (123, 124), and osteopontin (125–127), can also function as angiogenic factors (128–130). Other cytokines released by DCs that affect angiogenesis include IL-6 and TGFβ (129). Finally DCs can secrete pro-angiogenic chemokines such as CXCL1, CXCL2, CXCL3, CXCL5, CXCL8, and CCL2 (131–133).

On the contrary, mature cDCs can inhibit angiogenesis by releasing cytokines such as IL-12 (64, 77, 102), and angiostatic chemokines (CXCL9, CXCL10, and CCL21) (134). Mature pDCs can produce high amounts of the anti-angiogenic cytokine IFNα (78, 135, 136). Finally DCs can also produce anti-angiogenic ECM components including thrombospondin 1 (TSP) (137, 138) and long pentraxin-3 (PTX3) (139, 140) that regulate angiogenesis.

## Myeloid-Derived Suppressor Cells

Myeloid-derived suppressor cells represent another immune component that plays an active role in the “orchestration” of tumor promotion and immune evasion (141–143). These cells appear to be immature myeloid cells with features of both monocytes/macrophages and granulocytes. High levels of pro-inflammatory factors, such as GM-CSF, IL-1β, IL-6, and S-100 within the TUMIC induce recruitment and expansion of MDSCs, and enhance their pro-tumor activity (144, 145). Moreover, MDSCs are endowed with diverse and potent immuno-suppressive machinery on innate and adaptive immune effectors. Two mainly distinct MDSC subsets have been defined in both humans and mice with some differences: granulocytic MDSCs and monocytic MDSCs [reviewed in Ref. (141, 142)].

Interestingly in cancer patients these cells share several features and properties with progranulocytes or immature promyelocytes (146), and their blood levels correlate with clinical cancer stage, metastatic tumor burden, and are inversely correlated with clinical outcomes (146, 147). Noteworthy, MDSCs have a direct function in promoting tumor angiogenesis through releasing soluble factors, such as MMP9 and VEGF (Figure 1), and experimental data from mouse models suggest that they are also able to differentiate into ECs (21, 148).

Thanks to their high plasticity these cells can acquire diverse mechanisms for suppressing anti-tumor CD8^+^ T and natural killer (NK) cells. These include inducible forms of nitric oxide synthetase (NOS2) and arginase (ARG1), and by generating ROS (149–151). Further, MDSCs possess some common features with TAMs and TANs (5, 21). During hypoxia in the TUMIC, infiltrating mouse MDSCs have been shown to differentiate into TAMs (152), whereas MDSCs from lungs of tumor-free mice cultured with a tumor cell conditioned medium polarized into regDCs (101), further adding a new piece in the complex puzzle of the TUMIC society.

Myeloid-derived suppressor cells down-modulate naïve CD4^+^ and CD8^+^ T lymphocyte trafficking and re-circulation (153), inhibit CD8^+^ T cell tumor and tumor-draining lymph node infiltration (154), suppress NK cells (155, 156) and promote the conversion of naive CD4^+^ T cells into induced Tregs (157–159). It has also been shown that human monocytic MDSCs are able to produce TGFβ and retinoids thus supporting the trans-differentiation of Th17 cells into FOXP3^+^-induced Tregs (160).

Recently, a novel function for MDSCs as osteoclast progenitors has been reported, suggesting a direct involvement in the osteolysis process, a common complication in breast, lung, prostate carcinomas as well as multiple myelomas (Figure 1). Osteolytic lesions are associated with a poor prognosis (161). Interestingly, an abnormal expansion of a novel subset of MDSCs in peripheral blood of pediatric patients with metastatic sarcomas with features of fibrocytes as been also characterized (162). Fibrocytes are hematopoietic stem cell-derived fibroblast precursors that are involved in chronic inflammation, fibrosis, as well as wound healing.

## Natural Killer Cells

Natural killer cells are effectors lymphocytes of innate immunity that can potentially control tumors by their cytotoxic activity. Multiple human NK cells subsets have been found. The major subset is represented by CD56^dim^CD16^+^ NK cells that constitute about 90–95% of peripheral blood NK cells. The CD56^dim^CD16^+^ NK cells readily kill target cells upon proper recognition, and only briefly secrete high cytokine levels (163). In contrast, the CD56^bright^CD16^−^ NK cells (about 5–10% of peripheral blood NK cells) are poorly cytotoxic but produce large amounts of cytokines, including IFNγ, TNFα, and GM-CSF. Moreover, a third NK subset, decidual NK (dNK) cells, is found in the decidua (164) that are characterized by a CD56^superbright^CD16^−^ phenotype. This peculiar subset is able to release significant amounts of pro-angiogenic factors, in particular VEGF, PlGF, and IL-8, necessary for spiral artery formation during decidualization (164, 165). The low cytolytic activity of dNK cells appears to be involved in embryonic implantation to avoid a non-self rejection process.

Similarly to several other immune cells, NK cells can also infiltrate the tumor mass where they are apparently recruited. The TUMIC is also able to affect NK functionality by a wide array or cytokines and soluble factors, either inhibiting their cytotoxic function or promoting a pro-tumor/pro-angiogenic phenotype (Figure 1). The NK CD56^bright^CD16^−^ subset predominates in non-small cell lung cancer (NSCLC), exerting very low cytotoxicity on K562 tumor cells (166, 167). We have recently reported that tumor infiltrating NKs in NSCLC also produce elevated levels of VEGF, PlGF, IL-8 and induce, *ex vivo*, EC chemotaxis, and tube formation, recapitulating the angiogenic activity of the dNK subset (9).

Transforming growth factor beta is released in the decidua and by the TUMIC, and TGFβ appears to be able to polarize the peripheral cytotoxic CD56^+^CD16^+^ NK subset toward a CD56^bright^CD16^−^ subset with some characteristics of dNK cells (168, 169). We observed that TGFβ induced peripheral blood NK cells to produce angiogenic factors (9), suggesting that TGFβ may be involved in “flipping” the angiogenic switch of tumor infiltrating NKs, sustaining tumor progression (Figure 1).

There is mounting evidence that NK cells are involved in regulating metastatic dissemination. NK cells are often shown to reduce metastatic efficiency of tumor lines *in vivo* when using the experimental metastasis assay (170, 171). Recent studies on the metastatic process have suggested that the tumor micro- and macro-environments may play critical roles in metastatic dissemination [reviewed in Ref. (172, 173)]. One of the more novel concepts is that of the pre-metastatic niche, where the innate immune system plays a key role (41, 42, 174). Attenuation of NK cell activity is associated with generation of the pre-metastatic niche and metastasis efficiency in murine models (40), where conditioned media from hypoxic tumor cells was associated with increased NK recruitment and reduced NK cytolytic activity. MDSCs were also enhanced within the pre-metastatic niche and may play a key role in suppressing NK activity (40). A recent study has shown that the TAM family of tyrosine kinases are involved in NK-mediated attenuation of metastasis formation and that inhibition of their activity results in NK “licensing” to kill metastatic cells in murine models of in both hematogenous and lymphatic dissemination (175), suggesting potential clinical applications.

## T Cells

The inhibition of T lymphocytes influx during angiogenesis and tissue stroma remodeling represent a peculiar feature of the TUMIC, leading to impaired T cell functionality, due to activation and expansion of tumor-polarized myeloid cells (MDSCs, M2 macrophages, TAMs, and regDCs) as well as by soluble factors released by the tumor and by the associated-inflammatory cells. The typical immuno-suppressive environment of the TUMIC (176) is characterized by a strong commitment toward induction of CD4^+^CD25^+^FOXP3^+^ Treg polarization (103, 177), and/or Th2 and Th17 cell activation (160, 178). A novel mechanism by which Tregs could be directly involved in the tumor angiogenesis has recently been described (Figure 1). In ovarian cancer, hypoxia-induced angiogenesis, human, and mouse CD4^+^CD25^+^ Tregs secrete higher amounts of VEGFA (as compared to CD4^+^CD25^−^ T cells) and promote EC proliferation *in vitro* and *in vivo* (179). Although this pro-angiogenic effect could be indirect, the depletion of Tregs in ovarian tumor-bearing-mice correlated with a strong reduction of the VEGFA at the tumor site, suggesting a relevant role of Tregs in promoting tumor angiogenesis in ovarian cancer (179).

During interaction with DCs, activated CD4^+^ T cells can acquire neuropilin 1 (NRP1), a co-receptor that binds VEGF from DCs by an intercellular transfer mechanism (180). The resulting NRP1-expressing T cells bind DC-secreted VEGFA and could potentially behave as VEGF-carrying cells, promoting angiogenesis (Figure 1). Furthermore, Tregs selectively recruited and accumulated in the TUMIC by CCL22 and CCL28 secretion, constitutively express NRP1 (181), indicating a potential major role of these cells among others CD4^+^ T cell subsets in transferring additional VEGF to the tumor site (179).

T regulatory cells could also directly influence and trigger alternative activation of human monocytes displaying functions and phenotypes that mirrors M2-like TAMs (182). Interestingly, the shift from Th1 to Th2 immune microenvironment has been described during transition from precancerous to invasive stage in cervical carcinoma (183) and pancreatic cancers (184).

The Th1-type cytokine IFNγ, conventionally a potent anti-tumor and anti-angiogenic factor (84, 185–190) has also been shown to play a role in triggering MDSC immune-suppressive function together with other cytokines, such as IL-10 (149, 191, 192). However, the infiltration of memory cytotoxic CD8^+^ T lymphocytes and Th1 cells often correlate with good clinical outcome (193), but the origin and type of tumor plays a crucial role (194–196). Efficacious anti-tumor therapeutic or vaccine approaches tested in murine models resulting in protection from tumors are characterized by strong Th1 polarization, M1 macrophage activation, a TNFα response, and an anti-tumor IFNγ-producing CD8^+^ CTL (187, 188, 190, 197). Within the TUMIC, CD8^+^ T cells are conditioned to become CD8^+^FOXP3^+^ T regulatory cells with similar immuno-suppressive activities to that of CD4^+^ Tregs (198–200), adding more complexity to mechanisms by which the tumor polarization switch impairs immune responses. However, this newly described tumor immuno-regulatory T subset is a small part of the entire CD8^+^ T cell population and little is known concerning their role *in vivo* and clinical relevance in cancer patients (201).

## B Cells

The role of B cells in tumor initiation, progression, and angiogenesis is still debated. However, there is clinical evidence regarding their association with good prognosis of cancer patients and potential anti-tumor effect (202, 203). Several studies on mouse models suggest their involvement as inhibitory cells toward CD8^+^ CTL responses (204). Experimental data support a tumor-promoting role for B cells for skin sarcoma development, showing immunoglobulin deposition within the TUMIC (205) that was dependent on TNFα (206). The pro-tumor effect of antibody–antigen complexes was shown by knock out of FcγR receptors (207). Immune complex activation of these receptors on resident and recruited myeloid cells was also shown (208, 209). Interestingly, antigen–antibody complexes are involved in differentiation of M2c macrophages (8). Thus antigen–antibody complexes could influence the polarization of macrophages (Figure 1), as well as other immune cell types expressing Fc receptors (granulocytes, NK cells, DCs, MDSCs). It is not known if M2c macrophages have pro-angiogenic or even pro-tumor effects. However, these data also suggest that experiments performed using blocking antibodies must be interpreted with caution, even if proper isotype controls are used, since antigen–antibody complexes may influence many innate and even adaptive immune responses, including angiogenesis. B cell-derived lymphotoxin and activation of the NF-κB and STAT3 pathways has also been reported to promote prostate cancer progression (210).

Recently, a regulatory B cell subset (Bregs) has been characterized with pro-tumor function that shows inhibitory activities toward adaptive immune responses (208). However, the role of adaptive B cells in the tumor progression and angiogenesis is still not well understood and recently published controversial information highlights the need for further investigation (208, 209).

## Mast Cells

Mast cells represent a peculiar subtype of granulocytes found in peripheral tissue, which play a central role in inflammatory and immediate allergic reactions. Mast cells were initially suggested to be involved in vascularization during rheumatoid arthritis (211–214). They have also been found to be intimately involved in vascularization of hematological malignancies (215), where they are able to integrate into the vessel wall (Figure 1) by the process of vascular mimicry (216).

Mast cell contribution to the angiogenic switch in tumors is associated with the production of diverse angiogenesis-associated cytokines and chemokines (21). Proteases produced by mast cells promote pre-malignant angiogenesis (217–219) and are becoming a target for anti-angiogenic therapies (220, 221). Moreover, β-tryptase, a neutral serine protease that represents the most abundant mediator stored in mast cell granules, plays a crucial role in inflammation (Figure 1). β-tryptase release activates the protease-activated receptor type 2 that is directly involved in vascular relaxation and contraction (222). The pro-angiogenic activity of mast cells is enhanced by the interaction of their A_2B_ receptors with adenosine (223), released during tumor growth, tissue injury, ischemia, and inflammation. The interaction between adenosine and its A_2B_ receptor leads to secretion of VEGF, IL-8, and possibly other pro-angiogenic factors (Figure 1). The effect of these factors on new capillary formation is facilitated by the concomitant stimulation of mast cell A_3_ receptors that induce the expression of Ang2. These, and potentially others, factors released by mast cells act synergistically, and in a paracrine fashion, on ECs to induce angiogenesis.

## Cancer-Associated Fibroblasts

Fibroblasts are the most abundant cell type in connective tissues, forming the structural framework of tissues through their secretion of ECM components (224). Activated fibroblasts are directly involved in wound healing and fibrosis, both processes sharing a requirement for tissue remodeling. Since tumors are wounds that “do not heal” (225), the fibroblasts within the tumor mass, classified as cancer-associated fibroblasts (CAFs) contribute to the inflammatory orchestration of tumor angiogenesis (Figure 1). Interestingly, CAFs are of multiple origins: they can originate from resident fibroblasts, mesenchymal stem cells, or mutated fibroblasts (226). In this context, CAFs are able to produce cytokines and chemokines favoring immune cell infiltration, which in turn promotes angiogenesis and metastasis.

Stromal derived factor-1 producing pancreatic CAFs showed a synergy with IL-8 in the promotion of a complete angiogenic response (Figure 1) in recruiting ECs (227). Further, SDF-1 secreted by breast cancer CAFs has been involved in mobilization of endothelial precursor cells from bone marrow, favoring *de novo* angiogenesis, as well as in tumor growth through a paracrine effect on CXCR4-expressing cancer cells (228). CAFs are also able to produce CXCL14 in prostate cancer, this in turn enhances interactions with tumor cells and favor macrophages infiltration and M2 polarization (222). Recent studies reported that CAFs associated to incipient neoplasia are able to exhibit a pro-inflammatory signature, characterized by an over-expression of SDF-1, IL-6, and IL-1β that contribute to the recruitment of pro-angiogenic macrophages sustaining tumor growth (222).

## Concluding Remarks

Taken together, this view suggests that the host cells that constitute the TUMIC can be polarized to a pro-tumor phenotype characterized by a pro-angiogenic activity. Since the presence of these cells is critical for successful tumor growth to clinical relevance, they are therefore very interesting clinical targets. Clinical approaches in line with these findings, yet targeting only the VEGF pathway, have been recently approved, however, as shown here, there are many cell types and mediators to target. As our basic understanding of the mechanisms of host cell polarization is increasing and the effects of these phenomena become evident and appear to be key factors in tumor biology, it is necessary to find ways to prevent or revert these events, keeping tumors in a dormant, clinically indolent state.

## Conflict of Interest Statement

The authors declare that the research was conducted in the absence of any commercial or financial relationships that could be construed as a potential conflict of interest.
